# A survey of severe asthma in Canada: results from the CASCADE practice reflective program

**DOI:** 10.1186/s13223-024-00891-x

**Published:** 2024-04-18

**Authors:** Krystelle Godbout, Harold Kim, Irvin Mayers, James Paterson, Charles K. N. Chan

**Affiliations:** 1 Quebec Heart and Lung Institute, Laval University, Quebec City, Canada; 2 Division of Clinical Immunology and Allergy, Department of Medicine, Western University, London, ON Canada; 3 Department of Medicine, McMaster University, Hamilton, ON Canada; 4 Division of Pulmonary Medicine, Department of Medicine, Faculty of Medicine and Dentistry, University of Alberta, Edmonton, AB Canada; 5 Scientific Insights Consulting Group Inc., Mississauga, ON Canada; 6 Faculty of Medicine, University of Toronto, Toronto, ON Canada

**Keywords:** Severe asthma, Biological products, Treatment goals, Treatment satisfaction, Asthma phenotype, Standard of care

## Abstract

**Background:**

Since the last guidance was published by the Canadian Thoracic Society, there have been several advances in the clinical management of severe asthma. To gain a better understanding of the current standards of care and treatment patterns of patients, the CASCADE practice reflective program was established to conduct a real-world analysis of severe asthma management among specialists in Canada with a goal of identifying areas of opportunity to enhance patient management and outcomes.

**Methods:**

The CASCADE program was a two-part practice reflective and assessment program delivered through an on-line portal for selected specialists (Respirologists and Allergists) in Canada. The program consisted of a one-time overview survey of physician practice to establish overall practice parameters, followed by a review of at least 5 severe asthma patients to establish the current landscape of severe asthma management.

**Results:**

The program collected practice overview surveys from 78 specialists (52 Respirologists, 24 Allergists, and 2 General practice physicians with an interest in respiratory disease) in 8 provinces. Practices included a variety of types in both large metropolitan centres and smaller regional settings. There were 503 patients reviewed and included in the program. Most (65%) patients were currently using a biologic treatment, 30% were biologic naive, and 5% had used a biologic treatment in the past. Most patients (53%) were reported to have mixed allergic and eosinophilic phenotypes, despite a perception that allergic, eosinophilic and mixed phenotypes were evenly balanced in the physician practice. Overall, patients currently treated with biologic agents had parameters suggesting higher control and were more satisfied with treatment. However, there was less than optimal treatment satisfaction for more than half of all patients, particularly for those patients not treated with a biologic agent.

**Conclusions:**

Phenotyping is hampered by poor availability for several assessments, and the full range of treatments are not currently fully utilized, partly due to physician familiarity with the agents and partly due to prescribing restrictions. Even when treated with biologic agents, patient satisfaction can still be improved.

**Supplementary Information:**

The online version contains supplementary material available at 10.1186/s13223-024-00891-x.

## Background

Asthma is estimated to affect ~ 11% of the Canadian population, or nearly 250,000 Canadians [1] with long-standing estimates of ~ 5–10% of asthma patients with severe disease. It is a complex, heterogenous, and dynamic disease that is classified by treatment measures necessary for management: Severe asthma is defined by the Canadian Thoracic Society (and GINA) as requiring a high-dose inhaled corticosteroid and a second controller (or oral steroids) for six months or longer to maintain symptom control (or who do not achieve control with treatment) [2, 3]. In patients who remain uncontrolled despite optimized inhaled therapies, eligible patients may be considered for treatment escalation to biologic therapy. While clinical trials support the efficacy and safety profile of marketed biologics in Canada, real-world clinical experience is variable owing to the complexity and heterogeneity of disease and limitations related to the different clinical practice environments.

Since the publication of the last guidance document by the Canadian Thoracic Society in 2017 for the management of severe asthma, there have been major changes in the available treatment options, including the addition of a number of biologic agents, most often targeting individual downstream pathways or end products of type 2 inflammation [4] but also non-type 2 inflammatory pathways [5]. While severe asthma patients are typically managed primarily by primary care providers, those providers may not be qualified or comfortable to prescribe advanced therapies, so advanced management of patients with severe asthma frequently falls to specialists, despite the often slow referral of those patients to specialist care [6]. Effective management of asthma, including appropriate use of pharmaceutical agents, is an important consideration in the reduction of negative outcomes associated with inadequate asthma management. To improve the management of patients with severe asthma, identification of factors that hinder the timely use of advanced therapies in specialist care could aid in the development of practices to overcome these barriers.

To better understand the current standards of care and treatment patterns in the context of the growing complexity of severe asthma and expanding therapeutic options, we sought to evaluate current management practices in severe asthma through a practice reflective exercise conducted with specialist physicians treating severe asthma. We conducted an analysis of real-world severe asthma management among practicing specialists (Allergists and Respirologists) across Canada to gain insights into current management practices of physicians treating severe asthma. The overall purpose was to provide a current snapshot of management practices with the goal of identifying areas of opportunity to enhance optimal patient management and outcomes.

## Methods

The Canadian Asthma Specialists Collection and Discussion of Patient Experience (CASCADE) program was developed by a faculty consisting of Allergists and Respirologists from across Canada, with extensive experience in severe asthma. Program development was supported by the program sponsor and facilitated by an independent agency responsible for implementing the program. CASCADE was reviewed and accredited by an independent central research ethics board. The program was conducted from January to May 2023, available to physicians across Canada. The program was open to Canadian Allergists, Pneumologists/Respirologists or general practice physicians with a high proportion of patients with severe asthma in their clinical practice and who are authorized to prescribe biologic medications. Physicians were invited to participate after they were identified through routine interactions with representatives of the program sponsor.

The program consisted of two separate, physician completed surveys administered through a custom online portal. The first survey to be completed, consisting of 18 questions (see Additional file 1: Appendix 1), assessed the physician demographics (practice size, number of severe asthma patients, specialization, etc.) as well as their perceptions of their current practice (number of patients, distribution of phenotypes, percentage poorly controlled, etc.) and their general management of patients with severe asthma. The second survey was completed multiple times: once per severe asthma patient reviewed in the program that the physician had recently assessed in their clinical practice. Participating physicians were asked to review at least 5 patients using a survey designed to elicit their current choices for patient management and to provide a comparison to their overall perceptions of their own practice.

The patients that participating physicians were asked to assess were those who were aged 12 years or more, were diagnosed with severe asthma, had received high dose inhaled corticosteroid and at least one other maintenance medication. To try to reflect clinical practice, there were no specific exclusions for patients beyond age and diagnosis, however, physicians were encouraged to include patients that they had recently had a clinical visit with to reflect their most recent practice habits. All patient data was collected anonymously.

The overall goal of the program was to provide physicians with insights into their own practice, comparing their perceptions with their current, day-to-day practice habits, as well as allowing a comparison to the practice habits of their peers after aggregation of survey data through the program. To achieve this, the program was designed with a plan to aggregate all patient and physician surveys nationally, as well as aggregating the data provided for the patients collected by each individual physician. Summary statistics for each of the responses for each of the questions were prepared, and qualitative comparisons between individual physicians and national data were carried out, as well as between their perceptions and the patient-to-patient reality of their own practices.

Program delivery was facilitated using an independent organization with funding from the study sponsor. All editorial and data control resided with the steering committee. Ethical review and approval of the program was obtained from Center for IRB Intelligence/Advarra.

## Results

Overall, 503 patients were reviewed by physicians at 69 sites across Canada for the CASCADE program. A total of 78 physicians submitted the practice profile overview survey (9 physicians submitted the practice overview but did not review any patients for the program). Response rate for the program was high: 99 physicians were invited to participate, and 80% provided data for the program.

No specific strategy was employed for the selection of physicians to participate beyond ensuring representation from across Canada. In Canada, only specialists, specifically qualified internists or specifically qualified primary care physicians are authorized to prescribe biologics for asthma; all participating physicians were authorized to prescribed biologics. The majority (67%) of physicians were respirologists, with 2.6% of physicians reporting “other” specializations (internal medicine / general practice). The remaining (31%) reported allergy as a specialization; the ratio of respirologists to allergists in the program is similar to the national ratio. Physicians represented a broad distribution of experience and practice types from both larger metropolitan centres as well as smaller, regional urban centres, representing both academic and community practices. Physicians from all regions in Canada (Atlantic Provinces, Quebec, Ontario, Prairies, and the West), with the exception of the three territories, participated in the program. The program was available in both English (87% of physicians) and French (13% of physicians). Physician demographics are summarized in Table 1.

**Table 1:** Physician and practice demographic information (n = 78)

Number of physicians:	
Invited to participate	99
Submitted practice profile	78
Reviewed patients	69
Duration of practice, years	
Median	12
Range	1–46
Primary practice type, % (n)	
Academic or teaching hospital	37% (29)
Solo	26% (20)
Group	23% (18)
Community hospital	14% (11)
Specialization, % (n)	
Respirologist	67% (52)
Allergist	31% (24)
Other	3% (2)
Province of practice, % (n)	
Ontario	53% (41)
Quebec	18% (14)
Alberta	9% (7)
British Columbia	6% (5)
Nova Scotia	6% (5)
Newfoundland and Labrador	4% (3)
Saskatchewan	3% (2)
Manitoba	1% (1)
Estimated number of severe asthma patients seen in a week	
Median	5.0
Mean	8.3
Range	0–50
Mean (range) of physician estimated proportion of severe asthma patients in the practice who are:	
< 12 years old	2.5% (0–40%)
12–18 years old	6.2% (0–100%)
> 18 years old	91.3% (15–100%)

A total of 69 physicians reviewed at least one patient case that was included in the program. Of those, physicians reviewed an average of 7.3 patient cases (median 6) for the program. Patients included in the program had a median age of 55 years and physicians reported a median age of diagnosis of severe asthma of 35 years (Table 2). Comparison of patient age and patient age at diagnosis showed that nearly 1/3 (32%) of patients were diagnosed with severe asthma over 20 years prior, with nearly one in four (24%) reporting diagnosis in the last 2–5 years. Most patients (60%) were female.

**Table 2:** Patient demographic information (n = 503)

Patient sex, %	
Female	60%
Male	39%
Other	1%
Patient age	
Median, years	55
Range, years	12–93
Age at diagnosis	
Median, years	35
Range, years	1–84
Time since severe asthma diagnosis, %	
0–1 years	8%
2–5 years	24%
6–10 years	18%
11–20 years	17%
21 or more years	32%

### Asthma control

Physicians estimated that, on average, 31% of severe asthma patients in their practices were not controlled, with half of the physicians estimating that only ~ 25% or fewer of their patients are not well-controlled. Overall control was not reviewed in the program for the individual patient, however, a number of Canadian Thoracic Society control criteria were reviewed.

About 47% of patients reported 2 or more exacerbations requiring oral corticosteroids in the past 12 months and 14% of patients required hospitalization due to exacerbation in the past 12 months (Table 3). Overall, half (50%) of assessed patients experienced either 2 or more exacerbations or hospitalization in the past year (Table 3). The Asthma Control Questionnaire (ACQ) results were available for 40% of patients. In those patients with an available result, the mean score was 2.0, with 58% reporting a value of 1.5 or larger, the level considered to indicate poor asthma control [7, 8]. A further 19% of patients had results between 0.75 and 1.5, considered to be questionable control. While only 6% of patients had a sputum eosinophil test reported, 63% of those had sputum eosinophils above 3% and 38% with levels of 10% or greater. Taken together, 58% of patients reviewed in this program had at least one indicator (exacerbations, hospitalization, ACQ, sputum eosinophils) of poor asthma control.

**Table 3:** Patient data collected in patient review

	Never used a biologic (n = 152)	Biologic in the past (n = 26)	Currently using biologic (n = 325)	All patients (n = 503)
What asthma phenotype would you say this patient has?				
Allergic	11%	19%	13%	12%
Eosinophilic	18%	15%	29%	25%
Mixed	48%	58%	54%	53%
Non-type 2	20%	4%	4%	9%
Other	3%	4%	0%	1%
How many exacerbations requiring oral corticosteroids has the patient had in the past 12 months?				
None	28%	35%	45%	39%
1	15%	4%	14%	14%
2–3	46%	46%	31%	36%
More than 3	11%	15%	10%	11%
Has the patient been hospitalized in the past 12 months due to an asthma exacerbation?				
Yes	14%	15%	13%	14%
No	85%	81%	86%	86%
Unknown	1%	4%	0%	1%
What is the patient's latest ACQ score?				
0 to < 0.75	3%	4%	13%	9%
0.75 to < 1.5	5%	0%	9%	7%
1.5 or more	32%	27%	19%	23%
Not Available	60%	69%	60%	60%
What is the patient's latest Sputum Eosinophils?				
< 3%	2%	0%	2%	2%
3% to 10%	2%	4%	1%	1%
> 10%	1%	4%	2%	2%
Not available	95%	92%	95%	95%
What is the patient's latest FeNO?				
< 20 ppb	5%	8%	10%	9%
20 ppb or more	14%	19%	15%	15%
Not available	81%	73%	75%	77%
What is the patient's latest FEV_1_?				
Median % of predicted	70%	69%	78%	75%
Median Volume	2.0 L	2.0 L	2.2 L	2.1 L
Not available	9%	12%	7%	8%
What is the patient’s latest Skin Prick Test Result?				
Positive	55%	62%	60%	58%
Negative	14%	12%	13%	13%
Not available	31%	27%	27%	28%
What is the patient's latest absolute blood eosinophil count?				
Mean	354 cells/µL	379 cells/µL	479 cells/µL	436 cells/µL
< 150 cells/µL	29%	19%	30%	29%
150 to < 300 cells/µL	19%	19%	10%	14%
> 300 cells/µL	48%	58%	55%	53%
Not Available	4%	4%	4%	4%

### Other disease assessments

**Fig. 1:**
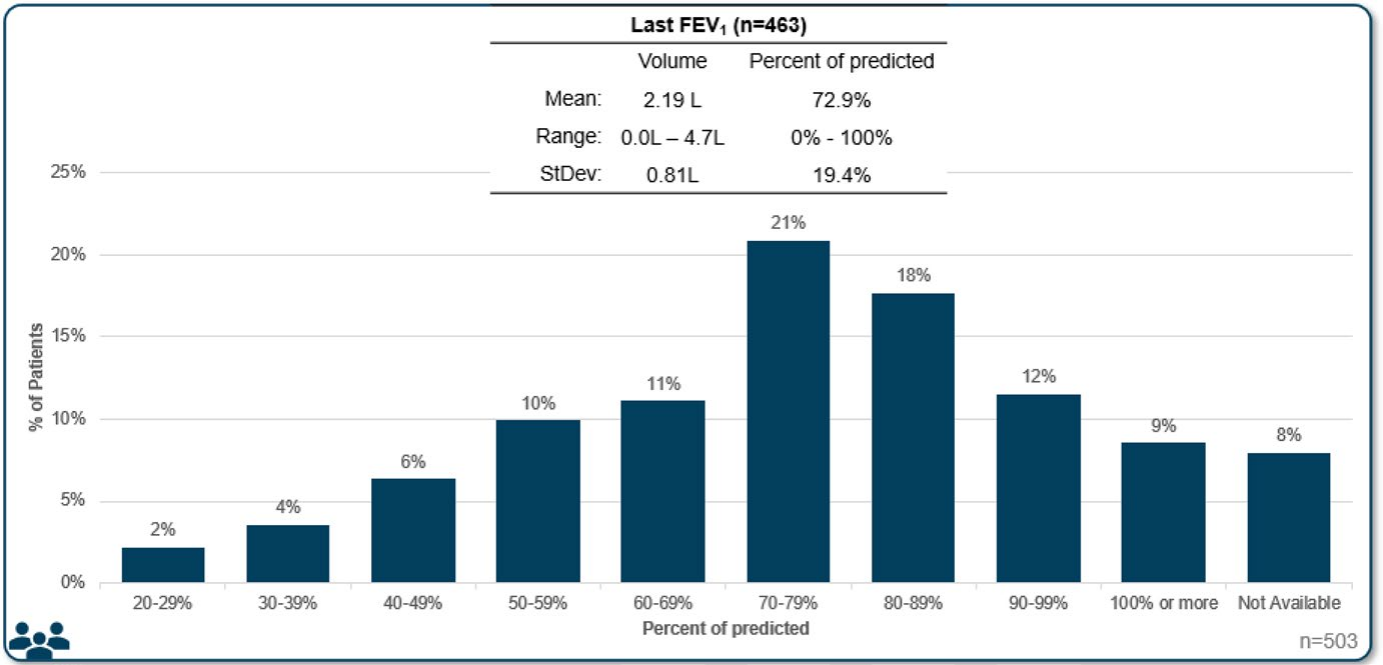
FEV_1_ results

**Fig. 2:**
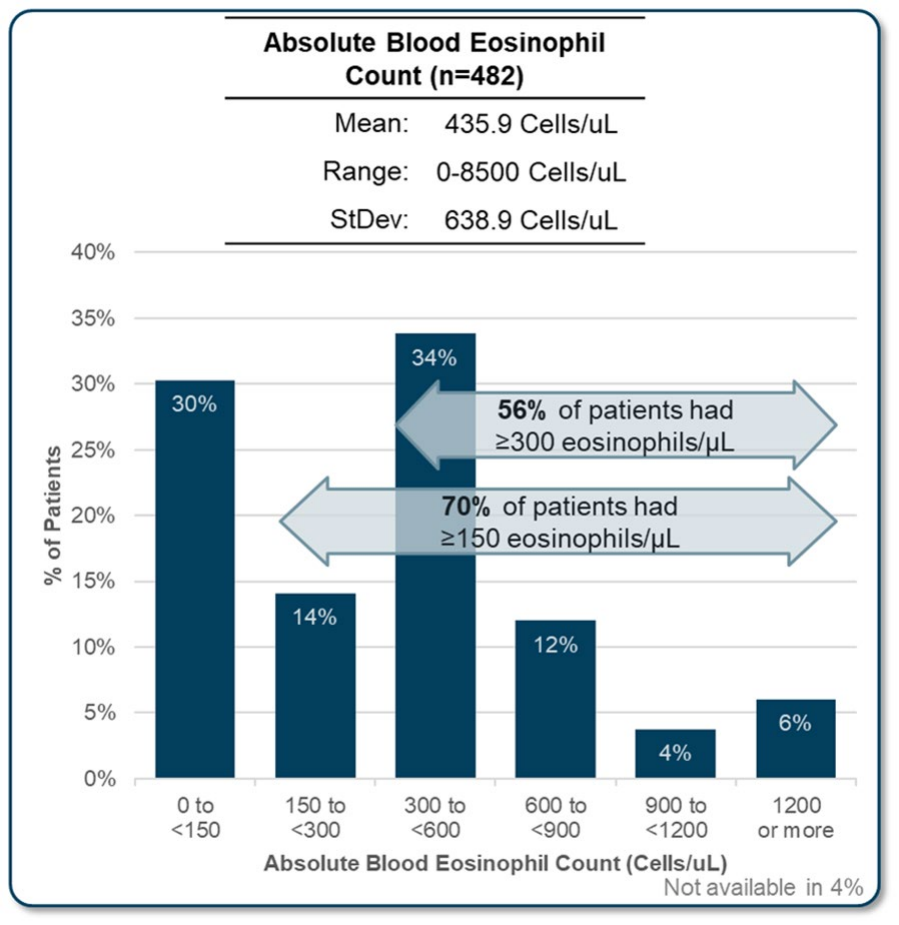
Most recent absolute blood eosinophil count for patients reviewed in the program

Despite the central importance of spirometry testing for diagnosis of asthma, FEV_1_ was not available for all patients reviewed. It was reported for 92% of all patients (79% in allergist practices, 98% in respirology practices). Median FEV_1_ was reported to be 72.9% of predicted, with a median volume of 2.1 L (Fig. 1, Table 3). Skin prick test results were also not completed for all patients, with results available for 97% of patients reviewed in allergist practices, compared to only 61% in respirology practices (72% total). In patients with a skin prick test, 82% were reported as positive. The specific panel for skin prick testing that was used was not asked, however, it is likely that most include both seasonal and perennial allergens. Blood eosinophil count was available for 96% of patents reviewed, with a mean of 436 cells/uL. More than three quarters (76%) of patients with an available result had an eosinophil count of 150 cells/uL or more and 56% had greater than 300 cells/µL (Fig. 2, Table 3). FeNO testing was available for 20% of the patient assessments, with 63% of available results being 20 parts per billion or greater.

### Biologic use

Nearly two thirds (65%) of patients assessed in the program were currently using a biologic; a further 5% had used a biologic in the past but were not currently using one (Table 3). The remaining 30% of patients had never used a biologic, a proportion similar to the physician’s reported perception of overall biologic use in their practice. Most (64%) physicians estimated that more than 61% of their patients were treated with biologic.

Control is impacted by biologic use. In patients that were currently using biologics, 54% of patients had at least one criterion suggesting poor asthma control (exacerbations, hospitalization, sputum eosinophils, or ACQ result), compared to 73% of patients who used biologics in the past and 66% of patients that never used biologics.

For those patients that had never used a biologic, eligibility was the most frequently cited factor for not using a biologic therapy (in 43% of patients). Other factors included patient reluctance in 18% of cases and 18% of reviews mentioned recent diagnosis or referral. Financial cost or reimbursement was a minor factor (7%). Physician perception was that reimbursement was the major barrier to biologic use (56% of physicians), with eligibility or patient not being a candidate cited as the major barrier by 23% of physicians. Patient reluctance was perceived by 19% of physicians as the major barrier. In the province of Quebec, which has a different reimbursement and eligibility framework, physicians perceived that eligibility or candidacy for biologic was the major barrier (57% of physicians) with patient reluctance and reimbursement cited by 21% of physicians as the major barrier. Review of Quebec patients not currently using a biologic revealed that eligibility was the primary barrier in 65% of patients, patient reluctance in 13% and other factors in 22%.

### Disease phenotype

**Fig. 3:**
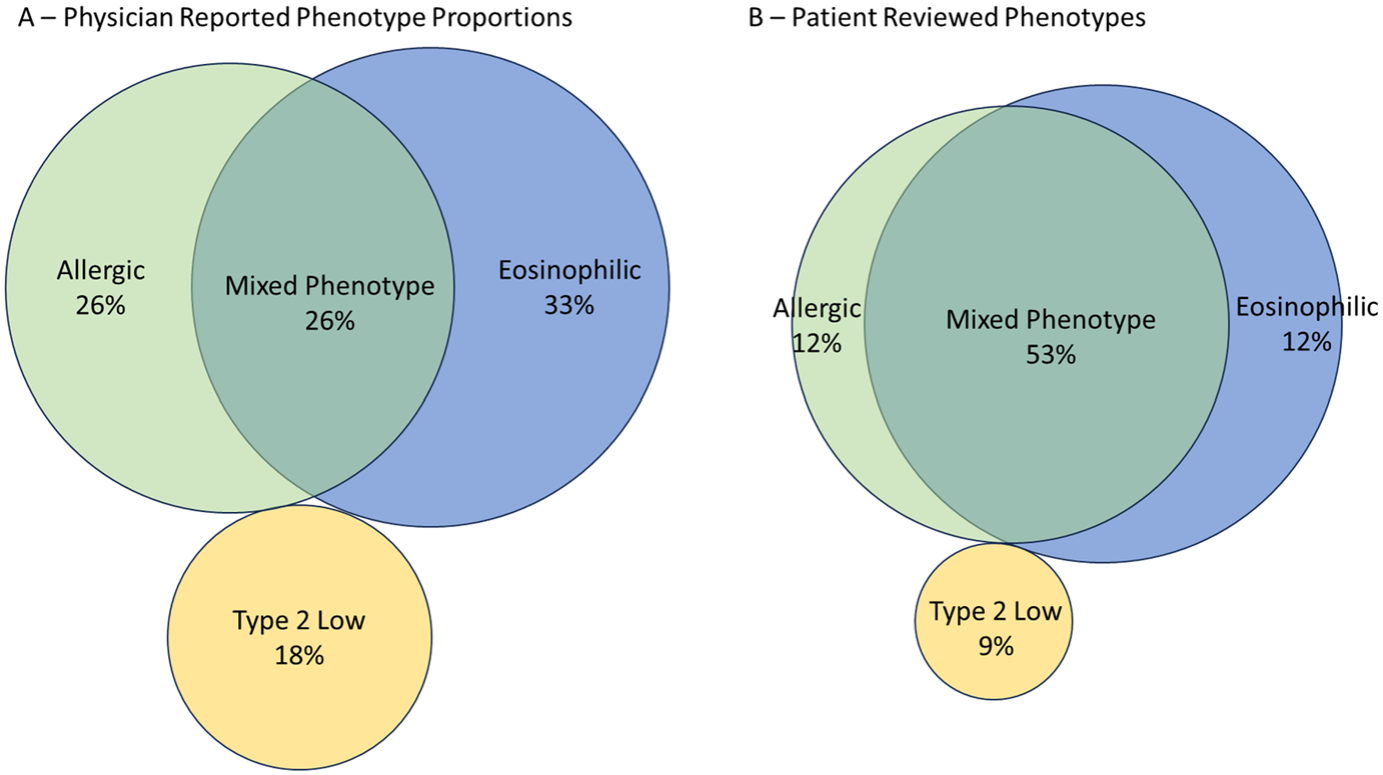
Venn diagrams of physician mean estimated phenotypic representations in practice (**A**) (n = 78) and the reported phenotypes of patients reviewed in the program (**B**) (n = 503). Mean proportion for each phenotype is reported

Physicians were asked to estimate the proportion of each asthma phenotype (mixed, eosinophilic, allergic, non-type 2, or other) in the severe asthma patients in their practice and were then asked the phenotype for each patient they included in the program. Definitions of each phenotype were not defined; physicians provided responses based on the definitions they apply to their own practice as this also reflects their clinical decision making. Overall, the estimated proportion of patients with each phenotype in the practice (Fig. 3a) did not match with the phenotype of the patients reviewed in the program (Fig. 3b, Table 3). Physicians perceived that a majority (33%) were eosinophilic, followed by allergic (26%), mixed (26%) and T2 low (18%) in their overall practice. Contrary to physician perception, review of the patients selected for the program revealed the mixed phenotype was actually the most predominant phenotype (53% of patients), followed by eosinophilic (25%), allergic (12%) and Type 2 low (9%).

### Treatment according to phenotype

**Fig. 4:**
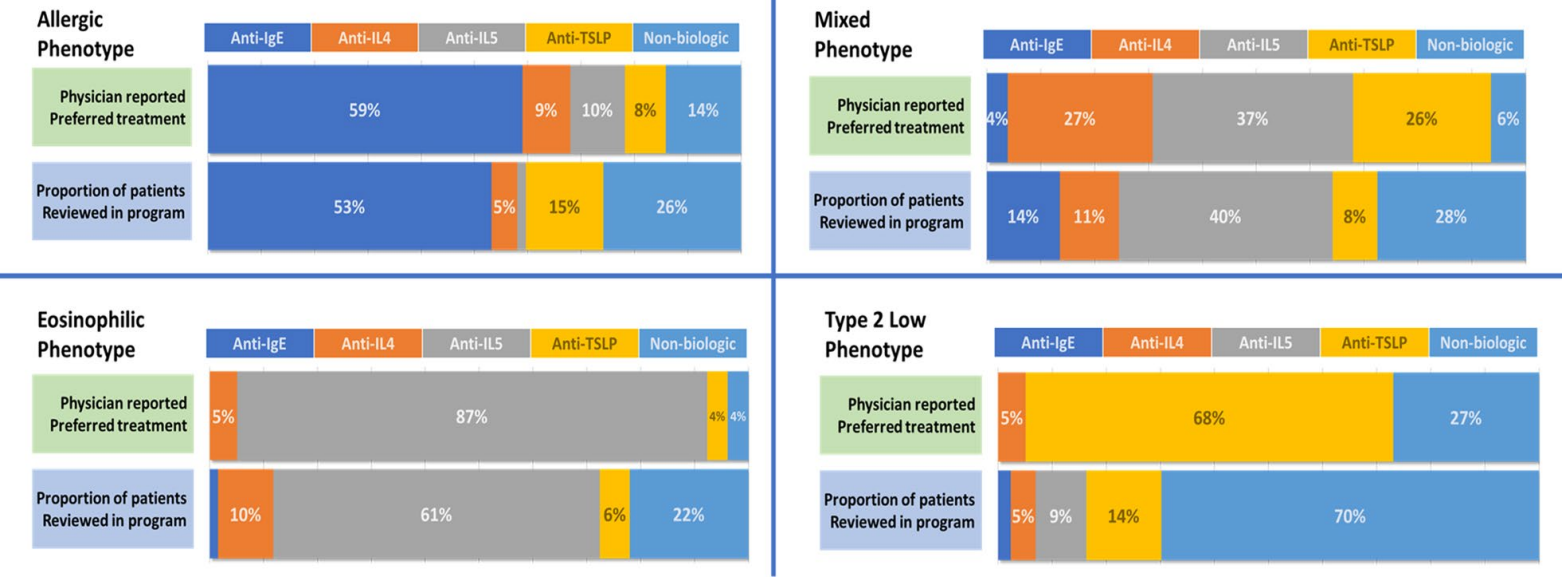
Physician reported preferred treatments (n = 78) for each severe asthma phenotype compared to patient prescribed (n = 503) treatment in the program. Physician preferences is the proportion of the number of physicians selecting each treatment as their preference compared with the entire group. Recorded treatment for patients assessed with allergic phenotype (n = 62), eosinophilic (n = 127), mixed (n = 265) or type 2 low (n = 42) were assigned to one of the four classes of biologic agents or to a treatment regimen that did not include a biologic

Physicians were questioned about their general treatment of choice for severe asthma in patients with certain phenotypes. Overall, most physicians indicated preferring an anti-IgE for allergic asthma (59% of physicians), an anti-IL5 for eosinophilic asthma (87%), and an anti-TSLP for Type 2 Low asthma (68%) (Fig. 4). For patients with mixed phenotype, physicians were divided between anti-IL4 (27%), anti-IL5 (37%) and anti-TSLP (26%). When compared to biologic use in patients reviewed, the pattern was maintained (Fig. 4). Most patients with an allergic phenotype were treated with an anti-IgE (53%), patients with eosinophilic asthma were treated with an anti-IL5 (61%), patients with mixed phenotypes were treated with a variety of agents, however, anti-IL5 agents were most frequently used (40%). Anti-TSLP and anti-IL5 were the most frequently used biologics for patients with type 2 low asthma (14% and 9%, respectively); however, most (70%) type 2 low phenotype patients were not treated with biologic agents. Reasons cited for the selected agents were most frequently biomarker with another factor for allergic or mixed phenotypes, or biomarker alone for eosinophilic phenotype.

### Treatment goals and satisfaction

**Fig. 5:**
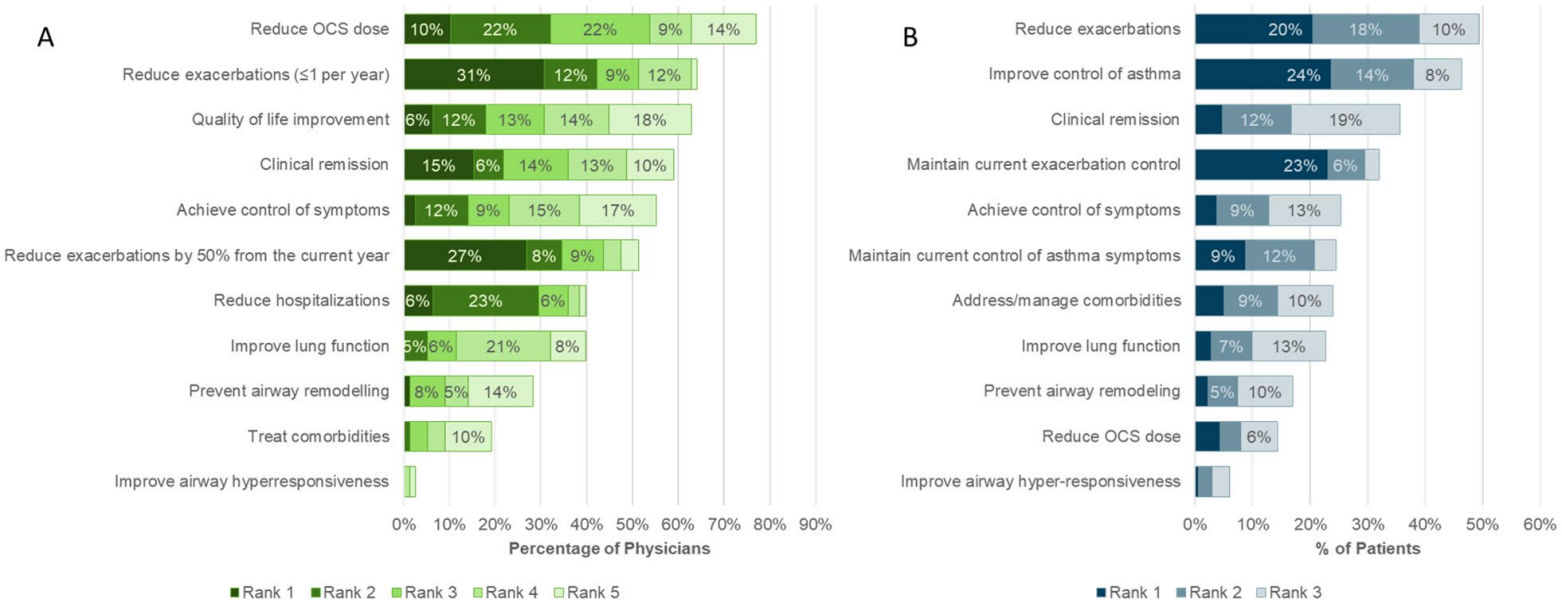
Treatment Goals for severe asthma. General overall treatment goals (**A**) and specific treatment goals for each patients assessed in the program (**B**)

When asked to select and rank the top five treatment goals for severe asthma patients in general, reduction in OCS use was the goal appearing most frequently in the top five (selected as a treatment goal by 77% of physicians) (Fig. 5A). However, exacerbation reduction was identified by physicians as the goal with the highest priority (reduce to less than 1 per year ranked first in 31% and reduce by 50% or more in 27%).

Physicians selected and ranked the top three treatment goals for each patient entered in the program. Exacerbation control or reduction and symptom control were most selected (Fig. 5B). Reduction in OCS was infrequently selected as a treatment goal for patient, suggesting that OCS use was already limited in these patients.

The majority of physicians (55%) agreed that reduction in symptoms of airway hyperresponsiveness should be considered a goal of therapy in severe asthma. Despite this, physicians rarely included it as one of the top 5 goals of therapy in general (3% of physicians), and it was not a prominent goal of therapy for the patients reviewed in the program, even though nearly two thirds of patients reported symptoms consistent with AHR.

**Fig. 6:**
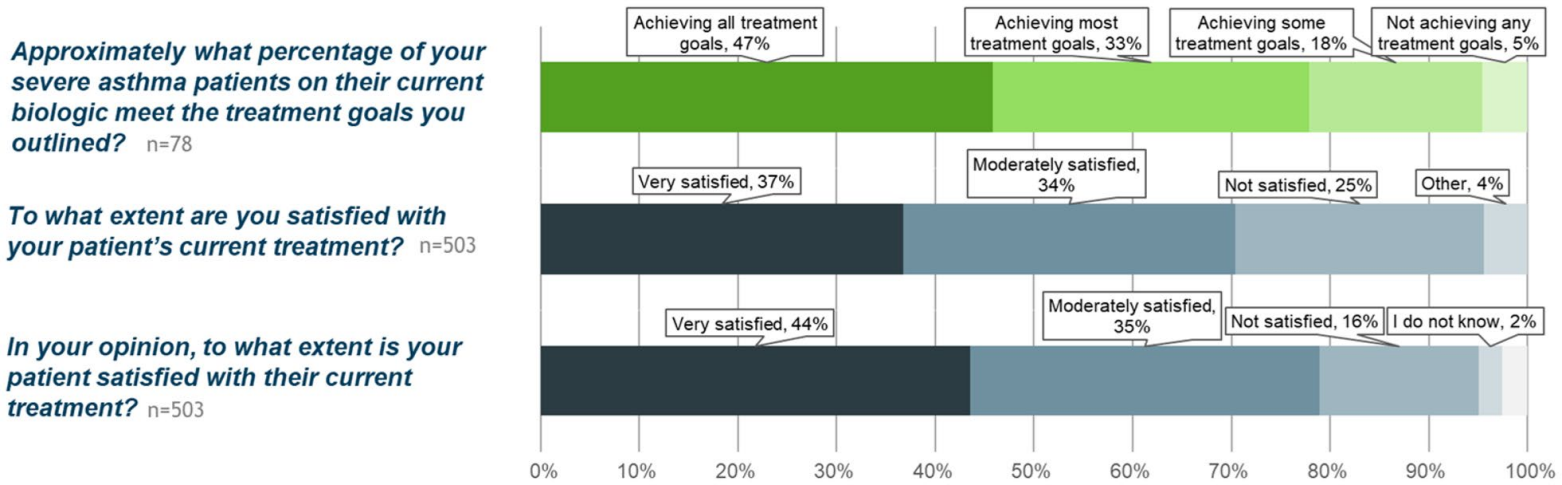
Treatment goals and satisfaction. Overall, physicians perceive that nearly half of their patients are meeting all treatment goals (top panel, green). In reality, physicians are fully satisfied and consider that their patients are meeting all goals in 37% (middle panel) and feel that patients are slightly more satisfied (bottom panel)

**Fig. 7:**
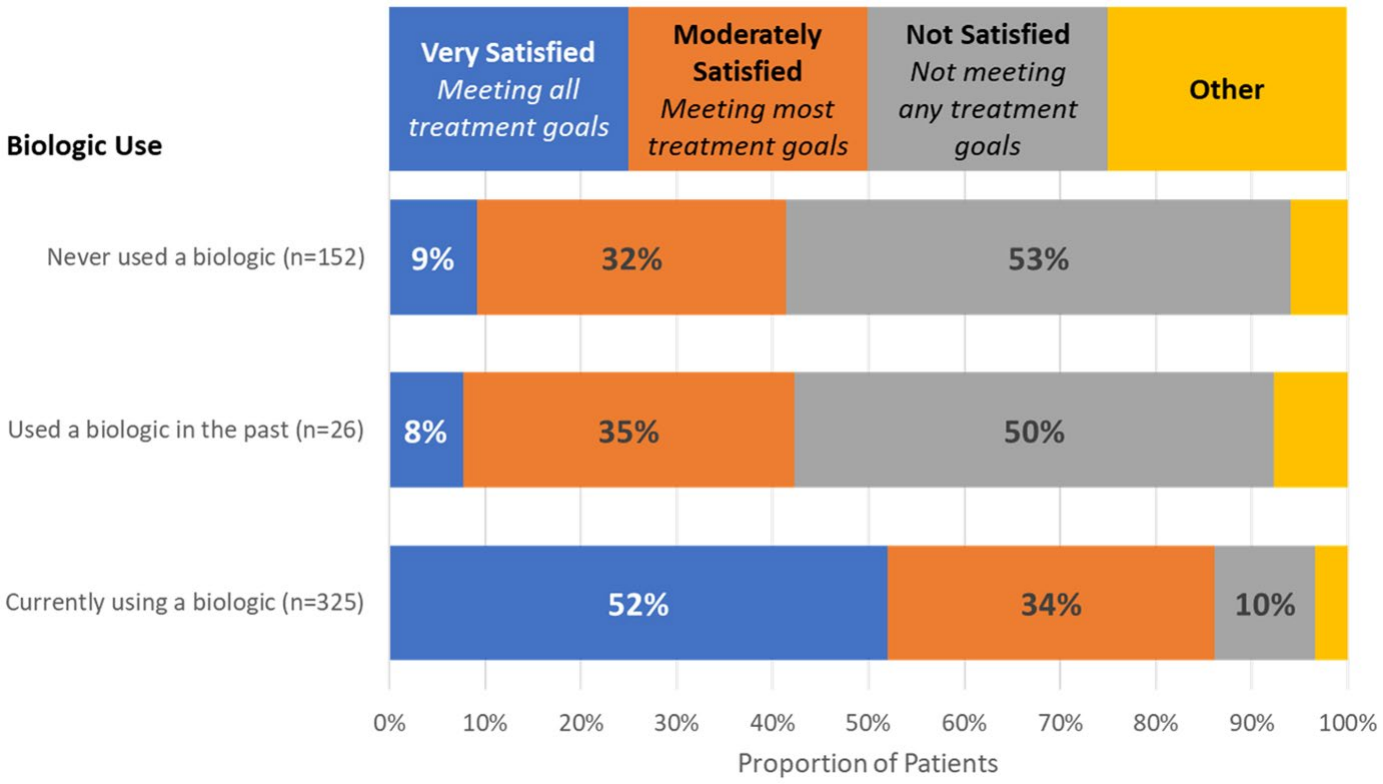
Physician satisfaction with treatment for assessed patients, based on biologic use

In general, physicians considered that a mean estimate of 47% of severe asthma patients in their practices were achieving all their treatment goals (Fig. 6). However, in specific patient assessments, physicians were fully satisfied with their patient’s treatment in only 37% of cases and thought that 44% of their patients were fully satisfied and meeting their treatment goals. In all cases, the majority of patients were not meeting all their treatment goals. When considering patient treatment with biologic, physicians were much more satisfied, with treatment for patients currently using a biologic when compared with patients who used a biologic in the past or who had never used a biologic (Fig. 7).

## Discussion

The overall objective of the CASCADE program was to allow physicians to assess their perceptions of their own clinical practice and then compare those perceptions with their clinical management of their own patients as well with the perception and clinical practice of their peers from across Canada. As part of the program, a series of meetings between the physicians who participated in the program were held, moderated by the program faculty, to identify barriers and gaps in patient management and to develop strategies to improve patient outcomes. The consensuses from these meetings will be published subsequently.

The physician practices included in the program were not specifically selected using any specific characteristic but through normal interactions with representatives of the sponsor based on an expressed interest in participation; sponsor representatives did not approach all physicians but did invite physicians with a wide variety of practice patterns. The practice profile data that were collected in the program support that the practices represented a diverse variety of clinical practices. Geospatial analysis of a variety of population characteristics from Canadian Census data (data not shown) suggested diverse patient populations for urban–rural ratio, large metropolitan centres versus smaller regional centres, a range of reported incomes and ethnic composition. Physician provided data further supports the diversity of the clinical populations: for example, physicians reported a varied proportion of children, youth or adult patients, with some practices reported a high number of children with severe asthma (up to 40%) and others reported no children (Table 1). Likewise, the number of patients seen in a typical week, practice type (academic versus community) and other data are consistent with a varied selection of physicians.

The program did not collect data on the total number of patients with any condition in the practice, nor did the program ask clinicians to specific any specific clinical interest. However, the program did ask physicians to estimate the proportion of their severe asthma patients who were adult, adolescent, or pediatric. It should be noted that this does not represent the distribution of patients that were included in the patient reviews as data collection was limited to adolescent and adult patients only. Overall, in these practices, an average estimate of 91% of severe asthma patients were adult (over the age of 18 years); however, there was variation between practices with some reporting higher estimates of younger patients. Considering the patients that were actually reviewed for this program, 97.4% where adult (over the age of 18 years), with only 2.6% adolescence patients.

This is the first Canadian assessment of specialist management of patients with severe asthma, particularly focusing on the use of biologic add-on therapies. Physician management of mild to moderate asthma [9] and in moderate to severe asthma [10], has been assessed following the release of GINA recommendations in 2021 [11], however, these were not focused on specialist care for severe asthma and biologic use. Perception of asthma control and management of patients from the Carenity asthma community was assessed in a study including 200 patients from 7 countries. However, this may not fully reflect Canadian patients as few patients from Canada were included [12].

Several key observations arose from this program. It identified that there is a difference in perception of the distribution of asthma phenotypes in specialist practice and the phenotype reported for individual patients, with patients of mixed phenotype representing a majority of patients (53%), while they are perceived to represent a much lower proportion (26%). The reason for this difference is unclear but may reflect either a selection of patients of mixed phenotype for inclusion in the program or an incorrect perception of the composition of their practice. The demographics of the patients included in this program were however similar to recent clinical trials in severe asthma (e.g., ASTHMA QUEST [13], NAVIGATOR [14], and others) suggesting that the included patients are representative of a severe asthma population.

Asthma phenotyping is considered an essential step in management of severe asthma [2], and is included in both the Asthma Canada patient charter [15, 16] and in the recommendations from the Canadian Delphi consensus for severe asthma [17]. However, the identification of phenotypes is complicated by the complexity of the underlying disease immunopathology, with current biomarkers representing an incomplete surrogate measure of underlying processes. Assessment of the biomarker data that was collected during the patient reviews shows that 83% of the patients had at least one biomarker associated with type 2 inflammation (FeNO above 20ppb, blood eosinophils 300 cells/µL or greater, sputum eosinophils 3% or greater, IgE of 30 or greater); when using a lower eosinophils threshold of 150cells/uL, the number of patients with at least one biomarker increases to 89%. This suggests that type 2 inflammation is the predominant mechanism for asthma pathogenesis in severe asthma and a common finding [18]. However, in this program, this may be due to a selection bias of the participating physicians who may have chosen patients on biologics as they are more easily identified but are also more likely to have a type 2 phenotype. Highly variable and mixed biomarkers are a reflect of real-world patient populations when compared with selected clinical trial populations. Physicians may perceive a limited number of biomarker driven phenotypes, but, in reality, the majority of patients will likely have characteristics found in many phenotypes. Analysis of the International Severe Asthma Registry identified that the majority of patients in the registry had multiple elevated biomarkers, with 59% having 2 or more [19]. An issue for effectively phenotyping asthma that has been confirmed in this study is poor access to specialized testing such as sputum analysis and FeNO testing [3, 17]. While FeNO was reported in about one quarter of the patients (24%), sputum eosinophils were infrequently available (6%). The expertise currently required to perform induced sputum analysis prevent its widespread use but point-of-care alternatives are being develop and may assist in future patient assessment [20].

In the Canadian context, there are a number of drivers that may impact specific assessments of asthma severity and phenotype. Typically, FeNO testing is not reimbursed by provincial medical systems and patients may not wish to pay for testing. FEV_1_ testing is essential, but in certain conditions it may also not be reimbursed (for example, when performed by allergists in Quebec). The underuse of testing for asthma represents a specific concern for ensuring that patients are offered the most effective treatment for their severe asthma.

A high proportion of patients in this study were experienced with biologic (65% currently using a biologic and 5% used a biologic in the past) which is consistent with studies performed in other jurisdictions. In the US, the CHRONICLE study identified that 66% of severe asthma subspecialist-treated US adults where using biologics [21]. However, data for proportion of patients using biologic treatments is scarce, and none could be identified for Canada. The data collected here offers an estimate of biologic use in Canada, but this must be tempered by the realization that the patients reviewed in this program were not rigorously selected and may therefore represent either an over- or under-representation of patients with severe asthma treated with biologics.

While biologic use was similar in Quebec versus the rest of Canada, there were different perceived and reported burdens for using biologics in the different jurisdictions. In Quebec, there was less consideration of cost or access for the use of biologics, but eligibility was a major barrier; in other provinces, the concern over cost and access was greater, although on the individual patient basis, that was not a limiting factor.

This program sought to assess treatment satisfaction and treatment goals, a different perspective from disease control that is assessed in clinical trials. It is clear that, for this group of specialists, there is a specific concern for overuse of systemic corticosteroids and the adverse outcomes associated with them [22]. These specialists, as a group, perceived that limiting OCS use was an important goal for patients with severe asthma, in general. However, when assessing patients on an individual basis, reduction in the OCS dose was infrequently cited, suggesting that this goal was already achieved for most patients entered in the program. While symptom and disease control is a well-established benefit of biologics in clinical trials, in real-world clinical settings, patient response to a specific biologic may be suboptimal and clinicians should be willing to explore other biologic therapies in their patients.

The program represents a point in time assessment of the patients included by the physician. It was clear that while patients using biologics were less likely to have frequent exacerbations (41% vs 57% of patients never using a biologic), the outcomes over time cannot be assessed. However, physicians indicated their intention to start a biologic for about half of the patients that were not currently using a biologic, most likely to address the high frequency of exacerbations. Indeed, 47% of the patients included in the program reported 2 or more exacerbations in the past year, a proportion that increased to 84% in those prescribed a biologic after the visit with the healthcare provider. Similarly, 68% of patients who were reported to be switching to a different biologic had experienced 2 or more exacerbations. It is however unclear if the starting or switching of biologic were triggered by these exacerbations or other factors of the clinical evaluation.

In the patients included in this program, when an ACQ value was available, it was generally indicating poor control. Over three quarters of patients had ACQ values that suggested poor or borderline control of asthma symptoms. However, it is understood, particularly by specialists treating severe asthma, that ACQ assessment may also capture non-asthma respiratory symptoms, leading to higher ACQ scores than those from asthma alone [23]. In some cases, the treating physician may consider that a patient is well controlled by current treatment despite an elevated ACQ value, particularly in those cases where the value is only slightly elevated. Hence, the high proportion of patient with ACQ above 1.5 in the program may be an overestimation of the real proportion of patients with symptomatic asthma.

Symptoms of airway hyperresponsiveness (AHR) were noted in the majority of patients assessed in this program. Furthermore, physicians reported that management of AHR should be considered a general goal of treatment. However, even though AHR is an important characteristic of the disease that contributes to disease pathology, this appears to be under-appreciated or disregarded in the individual patient assessments. This may be due to a relative lack of data regarding the impact of biologic treatment on AHR and a need to further investigate how to target this trait in clinical practice [24].

Patients were not deemed fully satisfied with their treatments, including those that were treated with biologics. This may reflect a biologic use that in poorly tailored to the patient’s disease. Biologic selection is an art based on the available evidence and the reimbursement requirements. However, the levels of individual biomarkers don’t always predict the response to the biologic treatment, and patients with variable levels may have stronger or weaker response [3]. Physicians should be cognizant of this factor and accept that patients with complex diseases may not respond to treatment in expected ways. Compounding this, biologics that have been commercially available for a longer time may be more frequently used, either because patients have been on these therapies for several years and are sufficiently satisfied or because physician are most comfortable with prescribing a drug they are familiar with. At the time of data collection for this program, tezepelumab had been available for prescription for less than one year, accounting for the limited use in this patient population.

While patients were not universally satisfied with their current biologic treatment, it was apparent from the program that patients currently using biologics were deemed much more satisfied with their treatment than patients who were not currently using a biologic (see Fig. 7). This suggests that biologics do improve patient goals. Due to limited sample size, it is unclear why the patients who used biologics in the past were no longer using them, but if those patients were achieving similar satisfaction when using a biologic, it is a wonder that they are not agitating for that treatment again.

In this program, more than 20% of patients reviewed (of any phenotype) where not using biologics, with 70% of patients with type 2 low phenotype not treated with biologics. These patients experienced much poorer satisfaction with treatment than those patients that were treated with biologics. In half of these cases, the major barrier for patients to biologic access was eligibility—likely referring to the specific criteria required for biologic prescription (exacerbations, biomarkers, OCS, etc.). The results from this program clearly show that patients not eligible to current biologics have an unmet need that should be addressed in future biologic development. Biologics effective in several phenotypes or in non-exacerbating patients may provide an avenue for these patients.

### Limitations

The CASCADE program was not comprehensive in the selection of practices to be included. Practice selection could be skewed by bias of both the recruiter and the physicians as not all physicians who were invited to participate in the program chose to do so. Further, the patient selection was not random or sequential and no specific criteria were given to the participating physicians to guide their selection. Selection of patients was rather left to the discretion of the physicians and their personal biases may have intruded, leading to a population that do not fully represent the makeup of patients in the practice. Because of these limitations, the findings of this program should be extended to other practices with care and these results should only be considered qualitatively.

The practice reflective program was carried out in the context of the Canadian single payer health care system and negotiated drug reimbursement criteria by Health Technology Access (HTA) organizations. We believe these approaches to align more closely to European Union models and less to the United States models of care. We hope to see similar programs carried out in other jurisdictions such as the European Union, the United States of America, or Asian Pacific countries.

## Conclusions

Despite guidelines advocating for phenotype specific biologic therapies, treatment patterns of biologics use in Canada do not always align well to patient phenotype. The majority of severe asthmatics in Canada present with a mixed phenotype yet are most commonly treated with biologics targeting individual downstream pathways of inflammation. The introduction of broader spectrum biologics and those acting farther upstream in the inflammatory pathway is relatively recent and experience with these agents is therefore more limited. In those patients with mixed phenotypes and multiple overlapping pathways of inflammation currently treated with narrow target biologics and who may not be achieving all their treatment goals, an upstream approach to biologic therapy may be advantageous in controlling more aspects of the underlying mechanism of disease. Based on this practice reflective program, it is clear that a significant proportion of patients are not meeting treatment goals on their current treatment regimen and changes to that regimen should be investigated. We hope that the results from this study can help guide specialists across Canada in further refining their approach in utilizing the right biologic for the right severe asthma patient to achieve optimal outcomes.

### Supplementary Information

**Additional file 1: Appendix S1**. Survey Questionnaires.

## Data Availability

The aggregate dataset used and analysed in during the current study are available from the corresponding author on reasonable request. Individual data are not publicly available to ensure anonymity of the individual patients reviewed for the program is maintained.

## Abbreviations

ACQ: Asthma control questionnaire
AHR: Airway Hyperresponsiveness
FeNO: Exhaled nitric oxide test
FEV1: Forced expiratory volume in 1 s
GINA: Global initiative for asthma
IgE: Immunoglobin E
IL4: Interleukin 4
IL5: Interleukin 5
ppb: Parts per billion
OCS: Oral corticosteroids
StDev: Standard deviation
TSLP: Thymic stromal lymphopoietin
