# Supplementary material for: A survey of severe asthma in Canada: results from the CASCADE practice reflective program
Source: Allergy Asthma Clin Immunol. 2024 Apr 18;20:31. doi: 10.1186/s13223-024-00891-x (PMC11027544; doi:10.1186/s13223-024-00891-x)
Supplement: Supplementary file 1 — Additional file 1: Appendix S1. Survey Questionnaires. [file 13223_2024_891_MOESM1_ESM.docx]

Appendix: Survey Questionnaires

**Practice Profile**

| In which province/territory do you currently practice? | Required; Select one |
| --- | --- |
| \| - Alberta \| - British Columbia \| - New Brunswick \| \| --- \| --- \| --- \| \| - Newfoundland and Labrador \| - Northwest Territories \| - Nova Scotia \| \| - Nunavut \| - Ontario \| - Prince Edward Island \| \| - Quebec \| - Saskatchewan \| - Yukon \| | |
| What are the first 3 digits of your practice postal code? | Required |
| \|  \| [Format of XNX] \| \| --- \| --- \| | |
| In which type of clinical practice do you work? | Required; Select one |
| \| - Academic/Teaching Hospital \| - Community Hospital \| - Group Practice \| \| --- \| --- \| --- \| \| - Solo Practice \| - Other; Please Specify: \|  \| | |
| What is your speciality? | Required; Select one |
| \| - Allergist \| - Respirologist \| \| --- \| --- \| \| - Other, Please Specify: \|  \| | |
| How many years have you been in practice? | Required; |
| \|  \| Years \| \| --- \| --- \| | |
| How many patients with severe asthma do you see in a typical week? | Required; |
| \|  \|  \| \| --- \| --- \| | |
| What percentage of those severe asthma patients do you consider uncontrolled? | Required; Enter Percent (0% to 100%) |
| \|  \| % \| \| --- \| --- \| | |
| Approximately what percentage of your patients with severe asthma are: | Required; Enter percent values (must sum to 100%) |
| \| Children (<12 years old): \| \|  0% \|  5% \|  10% \|  15% \|  20% \|  25% \|  30% \| \| --- \| --- \| --- \| --- \| --- \| --- \| --- \| \|  35% \|  40% \|  45% \|  50% \|  55% \|  60% \|  65% \| \|  70% \|  75% \|  80% \|  80% \|  90% \|  95% \|  100% \| \| \| --- \| --- \| --- \| --- \| --- \| --- \| --- \| --- \| --- \| --- \| --- \| --- \| --- \| --- \| --- \| --- \| --- \| --- \| --- \| --- \| --- \| --- \| --- \| \| Adolescents (12-18 years old): \| \|  0% \|  5% \|  10% \|  15% \|  20% \|  25% \|  30% \| \| --- \| --- \| --- \| --- \| --- \| --- \| --- \| \|  35% \|  40% \|  45% \|  50% \|  55% \|  60% \|  65% \| \|  70% \|  75% \|  80% \|  80% \|  90% \|  95% \|  100% \| \| \| Adults: \| \|  0% \|  5% \|  10% \|  15% \|  20% \|  25% \|  30% \| \| --- \| --- \| --- \| --- \| --- \| --- \| --- \| \|  35% \|  40% \|  45% \|  50% \|  55% \|  60% \|  65% \| \|  70% \|  75% \|  80% \|  80% \|  90% \|  95% \|  100% \| \| | |

| Approximately what percentage of your severe asthma patients would you estimate are of the following phenotype? | Required; Enter percent values (must sum to 100%) |
| --- | --- |
| \| Allergic Asthma: \| \|  0% \|  5% \|  10% \|  15% \|  20% \|  25% \|  30% \| \| --- \| --- \| --- \| --- \| --- \| --- \| --- \| \|  35% \|  40% \|  45% \|  50% \|  55% \|  60% \|  65% \| \|  70% \|  75% \|  80% \|  80% \|  90% \|  95% \|  100% \| \| \| --- \| --- \| --- \| --- \| --- \| --- \| --- \| --- \| --- \| --- \| --- \| --- \| --- \| --- \| --- \| --- \| --- \| --- \| --- \| --- \| --- \| --- \| --- \| \| Eosinophilic Asthma: \| \|  0% \|  5% \|  10% \|  15% \|  20% \|  25% \|  30% \| \| --- \| --- \| --- \| --- \| --- \| --- \| --- \| \|  35% \|  40% \|  45% \|  50% \|  55% \|  60% \|  65% \| \|  70% \|  75% \|  80% \|  80% \|  90% \|  95% \|  100% \| \| \| Type 2 low asthma: \| \|  0% \|  5% \|  10% \|  15% \|  20% \|  25% \|  30% \| \| --- \| --- \| --- \| --- \| --- \| --- \| --- \| \|  35% \|  40% \|  45% \|  50% \|  55% \|  60% \|  65% \| \|  70% \|  75% \|  80% \|  80% \|  90% \|  95% \|  100% \| \| \| Mixed Phenotype (Allergic & Eosinophilic): \| \|  0% \|  5% \|  10% \|  15% \|  20% \|  25% \|  30% \| \| --- \| --- \| --- \| --- \| --- \| --- \| --- \| \|  35% \|  40% \|  45% \|  50% \|  55% \|  60% \|  65% \| \|  70% \|  75% \|  80% \|  80% \|  90% \|  95% \|  100% \| \| \| Other (*optional*):   \|  \| \| --- \| \| *Required if other provided:*   \|  0% \|  5% \|  10% \|  15% \|  20% \|  25% \|  30% \| \| --- \| --- \| --- \| --- \| --- \| --- \| --- \| \|  35% \|  40% \|  45% \|  50% \|  55% \|  60% \|  65% \| \|  70% \|  75% \|  80% \|  80% \|  90% \|  95% \|  100% \| \| | |
| What proportion of your severe asthma patients have comorbidities? | Required; Enter percent for each (0% to 100% allowed) |
| \| Nasal polyps: \| \|  \| \| --- \| \| Atopic dermatitis: \| \|  \| \| --- \| \| \| --- \| --- \| --- \| --- \| --- \| --- \| \| Allergic rhinitis: \| \|  \| \| --- \| \| Chronic rhinosinusitis without nasal polyps: \| \|  \| \| --- \| \| \| Chronic spontaneous urticaria: \| \|  \| \| --- \| \| Eosinophilic esophagitis: \| \|  \| \| --- \| \| \| Obesity: \| \|  \| \| --- \| \| Food allergy: \| \|  \| \| --- \| \| \| Other:   \|  \| \| --- \| \| *If other specified:*   \|  \| \| --- \| \|  \|  \| | |
| Approximately what percentage of your current severe asthma patients on a high dose ICS/LABA require a biologic? | Required; Select one |
| \|  0-20% \|  21-40% \|  41-60% \|  61-80% \|  81-100% \| \| --- \| --- \| --- \| --- \| --- \| | |
| Of your patients currently eligible to receive a biologic, what percentage are currently treated with one? | Required; Select one |
| \|  0-20% \|  21-40% \|  41-60% \|  61-80% \|  81-100% \| \| --- \| --- \| --- \| --- \| --- \| | |

| What is the main reason why some bio-eligible patients in your practice do not receive a biologic treatment? | Required; Select one |
| --- | --- |
| \|  Patient is ineligible for biologics \|  Patient is not a candidate for biologics \| \| --- \| --- \| \|  Patient does not have coverage for biologics \|  Patient is reluctant to start on biologic \| \|  Other, Please specify: \|  \| | |
| What is your preferred treatment choice for each of the following phenotypes? | Required; Select one for each group |
| **Allergic asthma:** (*Required, Select one*)   \|  Anti-IgE (e.g. omalizumab) \|  Anti-IL4 (e.g. dupilumab) \| \| --- \| --- \| \|  Anti-IL5 (e.g. benralizumab, mepolizumab, reslizumab) \|  Anti-TSLP (e.g. tezepelumab) \| \|  Add additional inhaled maintenance (higher dose ICS or LAMA) \|  Oral corticosteroids \| \|  Macrolides (e.g. azithromycin, clarithromycin) \|  No preference \| | |
| **Eosinophilic asthma:** (*Required, Select one*)   \|  Anti-IgE (e.g. omalizumab) \|  Anti-IL4 (e.g. dupilumab) \| \| --- \| --- \| \|  Anti-IL5 (e.g. benralizumab, mepolizumab, reslizumab) \|  Anti-TSLP (e.g. tezepelumab) \| \|  Add additional inhaled maintenance (higher dose ICS or LAMA) \|  Oral corticosteroids \| \|  Macrolides (e.g. azithromycin, clarithromycin) \|  No preference \| | |
| **Mixed Phenotype (allergic & eosinophilic asthma):** (*Required, Select one*)   \|  Anti-IgE (e.g. omalizumab) \|  Anti-IL4 (e.g. dupilumab) \| \| --- \| --- \| \|  Anti-IL5 (e.g. benralizumab, mepolizumab, reslizumab) \|  Anti-TSLP (e.g. tezepelumab) \| \|  Add additional inhaled maintenance (higher dose ICS or LAMA) \|  Oral corticosteroids \| \|  Macrolides (e.g. azithromycin, clarithromycin) \|  No preference \| | |
| **T2 low asthma:** (*Required, Select one*)   \|  Anti-IgE (e.g. omalizumab) \|  Anti-IL4 (e.g. dupilumab) \| \| --- \| --- \| \|  Anti-IL5 (e.g. benralizumab, mepolizumab, reslizumab) \|  Anti-TSLP (e.g. tezepelumab) \| \|  Add additional inhaled maintenance (higher dose ICS or LAMA) \|  Oral corticosteroids \| \|  Macrolides (e.g. azithromycin, clarithromycin) \|  No preference \| | |
| What is the main reason why some bio-eligible patients in your practice do not receive a biologic treatment? | Rank 5 items, with 1 was more important and 5 as least important |
| \|  \| Reduce exacerbations (by 50% from the current year) \|  \| Reduce exacerbations (≤1 per year) \| \| --- \| --- \| --- \| --- \| \|  \| Reduce hospitalizations \|  \| Clinical remission (ie. living a normal quality of life while on asthma medications) \| \|  \| Reduce OCS dose \|  \| Improve lung function \| \|  \| Quality of life improvement \|  \| Improve airway hyperresponsiveness \| \|  \| Prevent airway remodelling/disease modification \|  \| Achieve control of symptoms (e.g., ACQ) \| \|  \| Treat comorbidities \|  \| Other, specify:   \|  \| \| --- \| \| | |

| Approximately what percentage of your severe asthma patients on their current biologic meet the treatment goals you outlined? | Required; Enter percent values (must sum to 100%) |
| --- | --- |
| \| Achieving all treatment goals: \| \|  0% \|  5% \|  10% \|  15% \|  20% \|  25% \|  30% \| \| --- \| --- \| --- \| --- \| --- \| --- \| --- \| \|  35% \|  40% \|  45% \|  50% \|  55% \|  60% \|  65% \| \|  70% \|  75% \|  80% \|  80% \|  90% \|  95% \|  100% \| \| \| --- \| --- \| --- \| --- \| --- \| --- \| --- \| --- \| --- \| --- \| --- \| --- \| --- \| --- \| --- \| --- \| --- \| --- \| --- \| --- \| --- \| --- \| --- \| \| Achieving most treatment goals: \| \|  0% \|  5% \|  10% \|  15% \|  20% \|  25% \|  30% \| \| --- \| --- \| --- \| --- \| --- \| --- \| --- \| \|  35% \|  40% \|  45% \|  50% \|  55% \|  60% \|  65% \| \|  70% \|  75% \|  80% \|  80% \|  90% \|  95% \|  100% \| \| \| Achieving some treatment goals but could do better: \| \|  0% \|  5% \|  10% \|  15% \|  20% \|  25% \|  30% \| \| --- \| --- \| --- \| --- \| --- \| --- \| --- \| \|  35% \|  40% \|  45% \|  50% \|  55% \|  60% \|  65% \| \|  70% \|  75% \|  80% \|  80% \|  90% \|  95% \|  100% \| \| \| Not achieving any treatment goals: \| \|  0% \|  5% \|  10% \|  15% \|  20% \|  25% \|  30% \| \| --- \| --- \| --- \| --- \| --- \| --- \| --- \| \|  35% \|  40% \|  45% \|  50% \|  55% \|  60% \|  65% \| \|  70% \|  75% \|  80% \|  80% \|  90% \|  95% \|  100% \| \| | |
| Should reductions in airway hyperresponsiveness be considered a goal/target for biologic therapies? | Required; Select one |
| \|  Yes \|  No \|  I'm not sure \| \| --- \| --- \| --- \| | |
| In your opinion, what are the barrier(s) in providing optimal management for severe asthma? | Required; Select all that apply |
| \| □ Proper asthma education \| □ Access to severe asthma centre of excellence \| \| --- \| --- \| \| □ Access to specialized tests like FeNO, sputum induction \| □ Coverage for biologics \| \| □ Other, Please specify:   \|  \| \| --- \| \|  \| | |

Patient Survey

| What is the age of the patient? | *Required; Enter number (12 to 120 allowed)* | |  |
| --- | --- | --- | --- |
| \|  \| Years \| \| --- \| --- \| | | |  |
| What is the sex of the patient? | *Required; Select one* | |  |
| \|  Male \|  Female \|  Prefer not to answer \| \| --- \| --- \| --- \| | | |  |
| At what age was this patient diagnosed with severe asthma? | *Required; Enter number (maximum 100)* | |  |
| \|  \| Years \| \| --- \| --- \| | | |  |
| What asthma phenotype would you say this patient has? | Required; Select one | |  |
| \|  Purely allergic \|  Purely eosinophilic \|  Mixed allergic/eosinophilic \| \| --- \| --- \| --- \| \|  T2 low \|  Other, Please specify:   \|  \| \| --- \| \|  \| | | |  |
| What comorbidities does this patient have, if any? | Required; Select all that apply | |  |
| \| □ Nasal polyps \| □ Atopic dermatitis \| □ Allergic rhinitis \| \| --- \| --- \| --- \| \| □ Chronic rhinosinusitis without nasal polyps \| \| □ Chronic spontaneous urticaria \| \| □ NSAID hypersensitivity \| □ Eosinophilic esophagitis \| □ Obesity \| \| □ Food allergy \| □ Other, Please specify   \|  \| \| --- \| \| □ None \| | | |  |
| In reviewing the patient chart, what is their latest, if available? | | | |
| IgE count | | *Required; Complete entry* | |
| \|  \| IU/ml \| □ Not available \| \| --- \| --- \| --- \| | | | |
| Absolute blood eosinophil count | | *Required; Complete entry* | |
| \|  \| Cells/uL \| □ Not available \| \| --- \| --- \| --- \| | | | |
| Skin prick test | | *Required; Complete entry* | |
| \|  Positive \|  Negative \|  NA \| \| --- \| --- \| --- \| | | | |
| Last FEV1 | | *Required; Complete entry* | |
| \|  \| L \|  \| % of predicted \| □ Not available \| \| --- \| --- \| --- \| --- \| --- \| | | | |
| Symptoms of airway hyper-responsiveness reported by the patient | | | |
| \|  Yes \|  No \| \| --- \| --- \| | | | |
| ACQ score | | | |
| \|  \|  \| □ Not available \| \| --- \| --- \| --- \| | | | |
| Methacholine or mannitol challenge | | | |
| \|  \| % \| □ Not available \| \| --- \| --- \| --- \| | | | |
| FeNO | | | |
| \|  \| ppb \| □ Not available \| \| --- \| --- \| --- \| | | | |
| Sputum eosinophils | | | |
| \|  \| % \| □ Not available \| \| --- \| --- \| --- \| | | | |
| Sputum neutrophils | | | |
| \|  \| % \| □ Not available \| \| --- \| --- \| --- \| | | | |
| Other test | | | |
| \|  \| Test \|  \| Result \| \| --- \| --- \| --- \| --- \| | | | |
| *If either IgE or blood eosinophil values entered above:*  Which pathway are you choosing to address IgE and eosinophils? | *Required; Select one* | |  |
| \|  Allergic asthma \|  Eosinophilic asthma \|  Overlap allergic/eosinophilic asthma \| \| --- \| --- \| --- \| \|  No values for IgE and eosinophils available \| \|  \| | | |  |
| How many exacerbations requiring oral corticosteroids has the patient had in the past 12 months? | *Required; Select one* | |  |
| \|  0 \|  1 \|  2 \|  3 \|  >3 \| \| --- \| --- \| --- \| --- \| --- \| | | |  |
| Has the patient been hospitalized in the past 12 months due to an asthma exacerbation? | *Required; Select one* | |  |
| \|  Yes \|  No \|  I don't know \| \| --- \| --- \| --- \| | | |  |
| Has your patient ever been on a biologic? | *Required; Select one* | |  |
| \|  Yes, they are currently on a biologic \| \| --- \| \|  Yes, they were previously on a biologic, but discontinued   \| *If discontinued selected:*   \|  Lost financial coverage/too expensive for patient \|  Was not effective/Did not achieve treatment goals \| \| --- \| --- \| \|  Patient concerns about safety \|  Other, Please specify:   \|  \| \| --- \| \| \| \| --- \| --- \| --- \| --- \| --- \| --- \| \| \|  No, they have never been on a biologic   \| *If No selected:*   \|  No financial coverage/too expensive for patient \|  Not eligible for a biologic (does not meet criteria) \| \| --- \| --- \| \|  Patient reluctance \|  Patient concerns about safety \| \|  Other, Please specify:   \|  \| \| --- \| \|  \| \| \| --- \| --- \| --- \| --- \| --- \| --- \| --- \| --- \| \| | | |  |

| *If patient is using or has used a biologic:*  What class of biologic were/are they on? | *Required; Select one* |
| --- | --- |
| \|  Anti-IgE (e.g. omalizumab) \|  Anti-IL4 (e.g. dupilumab) \| \| --- \| --- \| \|  Anti-IL5 (e.g. benralizumab, mepolizumab, reslizumab) \|  Anti-TSLP (e.g. tezepelumab) \| | |
| *If patient is using or has used a biologic:*  Why was this treatment chosen for this patient? | *Required; Select one* |
| \|  Driven by biomarker and another factor (e.g. comorbidity) \|  Driven solely by a particular biomarker (if yes which one)   \|  IgE \|  EOS value \|  FeNO \|  Other, Please specify:   \|  \| \| --- \| \| \| --- \| --- \| --- \| --- \| --- \| \| \| --- \| --- \| --- \| --- \| --- \| --- \| --- \| \|  Driven by provincial or insurance reimbursement rules \|  Other, Please specify:   \|  \| \| --- \| \| | |
| What are your top 3 current goals for this patient? | *Rank 3 items, with 1 as most important and 3 as least important* |
| \|  \| Maintain current exacerbation control \| \|  \| Maintain current control of asthma symptoms \| \| \| --- \| --- \| --- \| --- \| --- \| --- \| \|  \| Address/manage comorbidities \| \|  \| Improve control of asthma \| \| \|  \| Reduce exacerbations \| \|  \| Prevent airway remodeling \| \| \|  \| Clinical remission (E.g. living a normal quality of life while on asthma medications) \| \| \| \| \| \|  \| Improve airway hyper-responsiveness \| \|  \| Achieve control of symptoms \| \| \|  \| Reduce OCS dose \| \|  \| Improve lung function \| \| \|  \| Other, specify: \|  \| \| \|  \| | |
| To what extent are you satisfied with your patient’s current treatment? | *Required; Select one* |
| \|  Very satisfied – meeting all goals \|  Moderately satisfied – doing as well as can be expected for current treatment \|  Not satisfied \|  Other, Please Specify:   \|  \| \| --- \| \| \| --- \| --- \| --- \| --- \| --- \| | |
| In your opinion, to what extent is your patient satisfied with their current treatment? | *Required; Select one* |
| \|  Very satisfied: it is meeting all their treatment goals for this patient \|  Moderately satisfied: it is meeting some of their treatment goals, but there is room for improvement \|  Not satisfied: they seem dissatisfied with the current treatment \| \| --- \| --- \| --- \| \|  I do not know \|  Other, Please Specify:   \|  \| \| --- \| \|  \| | |
| What was your course of action today? | *Required; Select one* |
| \|  Remain on current treatment, no change today \|  Initiate a biologic \|  Switch to a different biologic \| \| --- \| --- \| --- \| \|  Couldn’t switch due to coverage restrictions \|  Make adjustments to other medications (e.g. inhalers, leukotrienes antagonist) \|  Other, Please Specify:   \|  \| \| --- \| \| | |
